# PRESIDENTIAL SYMPOSIUM: FRAMING, BRIDGING, AND TRAVERSING SOCIAL DETERMINANTS OF HEALTH, DISPARITIES, AND INEQUITIES IN OLDER ADULTS

**DOI:** 10.1093/geroni/igad104.0799

**Published:** 2023-12-21

**Authors:** Deb Bakerjian, Mo-kyung Sin

## Abstract

In alignment with the theme of this year’s conference, Building Bridges>Catalyzing Research>Empowering All Ages, this symposium focuses on research that frames, bridges, and transverses various constructs of the social determinants of health, intersectional frameworks, and inequities that impact older adults at various times in life from mid to older adulthood through death. We start with a framework for how to consider social and structural conditions in which people live and the cumulative effects that an individual’s life course might impact their trajectory with Alzheimer’s disease and other related dementias and their families. Continuing on with the theme of family, dementia, and caregivers, we delve into the differences in caregiver burden across and within gender and racial/ethnic groups, looking at the linkage with financial, emotional, and physical burdens. From there, we go on to explore whether there is an association between negative family interactions and mental health outcomes and discuss how those outcomes might vary by race and ethnicity. Building upon several concepts in these first three papers, we will discuss how one can evaluate the intersectional impact of race and gender on end-of-life outcomes including pain, anxiety/depression, patient autonomy, and overall care quality. Our last speaker will share intriguing, and somewhat unexpected findings related to social inequities on health from midlife to death and the impact of early exposure to socioeconomic disadvantages might have on disparities. Given the importance of reducing inequities to improve health outcomes, this HS section Presidential Symposium is sure to challenge and enhance our thinking.

